# Proximity to risk-appropriate perinatal hospitals for pregnant women with congenital heart defects in New York state

**DOI:** 10.1186/s12884-020-03025-4

**Published:** 2020-06-01

**Authors:** Lauren E. Schlichting, Tabassum Insaf, George Lui, Ali Zaidi, Alissa Van Zutphen

**Affiliations:** 1 Hassenfeld Child Health Innovation Institute, Brown University, 121 S Main Street, Providence, Rhode Island RI 02912 USA; 2 Bureau of Environmental and Occupational Epidemiology, New York State Department of Health, Albany, NY USA; 3 University at Albany School of Public Health, Rensselaer, NY USA; 4 Pediatric Cardiology and Cardiovascular Medicine, Lucile Packard Children’s Hospital Stanford, Stanford, California USA; 5 Albert Einstein College of Medicine, The Bronx, NY USA; 6 Children’s Hospital at Montefiore, Bronx, New York USA; 7 Sinai Adult Congenital Heart Disease Program, Icahn School of Medicine at Mount Sinai, New York, NY USA

**Keywords:** Adult congenital heart disease, Pregnancy, Access to care, Disparities, Spatial analysis

## Abstract

**Background:**

Women with congenital heart defects (CHDs) experiencing pregnancies require specialized delivery care and extensive monitoring that may not be available at all birthing hospitals. In this study, we examined proximity to, and delivery at, a hospital with an appropriate level of perinatal care for pregnant women with CHDs and evaluated predictors of high travel distance to appropriate care. Appropriate care was defined as Level 3 perinatal hospitals and Regional Perinatal Centers (RPCs).

**Methods:**

Inpatient delivery records for women with CHD in New York State (NYS) between 2008 and 2013 were obtained. Driving time and transit time were calculated between the pregnant woman’s residence and the actual delivery hospital as well as the closest Level 3 or RPC hospital using Geographic Information Systems (GIS). Linear and logistic regression models evaluated predictors of high distance to, and utilization of, appropriate delivery care respectively.

**Results:**

From 2008 to 2013, there were 909 deliveries in a NYS hospital by women with CHDs. Approximately 75% of women delivered at a Level 3 or RPC hospital. Younger women, those who reside in rural and smaller urban areas, and those who are non-Hispanic White had a greater drive time to an appropriate care facility. After adjustment for geographic differences, racial/ethnic minorities and poor women were less likely to deliver at an appropriate delivery care center.

**Conclusions:**

Although most women with CHDs in NYS receive appropriate delivery care, there are some geographic and socio-demographic differences that require attention to ensure equitable access.

## Background

More women with congenital heart defects (CHDs) are reaching childbearing age due to improved survival resulting from advancements in treatment and management. However, pregnancy places an enormous strain on the circulatory system and women with CHDs often require additional monitoring and care throughout pregnancy and delivery due to their condition [1, 2]. Maternal heart disease is a major cause of maternal death during pregnancy and congenital heart disease is the most frequent cardiovascular disease present during pregnancy in the western world (75–82%). Depending on the severity of their defect and the presence of other comorbidities, pregnant women with CHDs may require access to specialty care centers and hospitals with high levels of obstetric care to prevent maternal and neonatal complications [3, 4]. Therefore, the American College of Cardiology and the American Heart Association recommend that pregnant women with complex, high-risk CHDs deliver at specialty care centers with maternal-fetal medicine subspecialists [1]. There has been no published research that investigates whether pregnant women with CHDs are delivering at their nearest hospital or require traveling farther to a hospital with specialized services. It is important to understand how travel distance may impact access to appropriate care, especially for women with health conditions that may complicate delivery. The aim of this study is to examine proximity to, and delivery at, a hospital with an appropriate level of perinatal care for pregnant women with CHDs and evaluate predictors of high travel time, via personal and public transportation, to appropriate care.

## Methods

This research was conducted as part of a larger population-based project on surveillance of congenital heart defects among adolescents and adults with methods described previously [5]. For this analysis, inpatient data from the New York State Statewide Planning and Research Cooperative System (SPARCS) for the years 2008 to 2013 were analyzed for women of childbearing age (15–44 years old). The SPARCS database contains hospital inpatient and outpatient discharge data including individual level data on patient characteristics, diagnoses and treatments, services, and facility information [6].

To identify individuals with CHDs in SPARCS, ICD-9-CM codes 745.x-747.x were used, based on the CHD eligibility definition developed specifically for the surveillance project [5]. Patient records with ICD-9-CM codes 746.86 (congenital heart block), 747.32 (pulmonary arteriovenous malformation/aneurysm), 747.5 (absence or hypoplasia of umbilical artery), 747.6x (other anomalies of peripheral vascular system), or 747.8x (other specified anomalies of circulatory system) in isolation were excluded from the CHDs case cohort, as these malformations are considered as part of the greater circulatory or peripheral vascular system, rather than defects of the heart. A five category CHD ICD-9-CM code classification scheme developed for the surveillance project based on the classification system of Marelli et al. (2007) was used to assign women to a single severity category: severe defect, shunt + valve defects, shunt defect only, valve defect only, and other defect (Supplementary Table 1) [5, 7, 8]. Further classification provided three groups for analysis: severe CHD, mild-to-moderate CHD, and isolated atrial septal defect (ASD). The severe CHD group contains women with an ICD-9-CM code in the severe category of the classification system. The mild-to-moderate CHD group contains women within the shunt, valve, shunt and valve, and other categories of the classification system, excluding those with an isolated 745.5 ICD-9-CM code, who represented the isolated ASD group. As isolated ASDs are a milder form of CHDs and the code used often includes patent foramen ovale (PFO), they were placed in a separate, isolated group.

ICD-9-CM Diagnosis-related group (DRG) delivery codes were selected to identify vaginal (767, 768, 774, and 775) and cesarean (765, 766) delivery hospitalizations using an enhanced delivery identification method [9]. Hospitalizations of parturient women for delivery were also identified using ICD-9-CM codes for normal delivery (650), outcome of delivery (V27), and diagnosis codes for pregnancy and/or labor complications (640.0–676.9, where the fifth digit is 1 or 2). Delivery related procedure codes 72.x, 73.x, and 74.x (excluding 74.91) were also utilized to identify delivery hospitalizations.

Comorbidities were examined using the Clinical Classifications Software (CCS) tool developed by Agency for Healthcare Research and Quality [10] and categorized into 24 broad comorbidity groupings as described previously [5]. For this analysis of pregnant women, comorbidities were further collapsed into two groups, cardiovascular and other. A woman was classified as having a cardiovascular related comorbidity if she possessed at least one of the following comorbidities: systemic hypertension, congestive heart failure, coronary artery disease, conduction disorder, venous disorder and phlebitis, hyperlipidemia, or other cardiovascular conditions. Other comorbidities examined included respiratory and pulmonary conditions, non-CHD birth defects, diabetes mellitus, other endocrine conditions, mental health conditions, neurologic and central nervous system conditions, musculoskeletal conditions, injury or trauma, immunology/rheumatology/allergy conditions, infectious diseases, hematologic conditions, neoplasms, gastrointestinal issues, renal, and other genitourinary and gynecological conditions.

Hospital levels for perinatal care were assigned using the New York State Department of Health (NYSDOH) regionalized perinatal services classification system as established under NYS regulation 10 CRR-NY 721.9 [11]. The current hierarchal system contains four levels of perinatal care, with Level 4 hospitals, also known as Regional Perinatal Centers (RPCs), providing the highest level of care to pregnant women and newborns. Level 1 Perinatal Centers provide care to normal and low-risk pregnant women and newborns; Level 2 Perinatal Centers provide care to women and newborns at moderate risk; Level 3 Perinatal Centers provide care for patients requiring increasingly complex care; Regional Perinatal Centers provide the highest level of care [11, 12]. All but Level 1 Perinatal Centers operate neonatal intensive care units. For the purposes of this study, Level 3 and RPC hospitals were used to define facilities capable of providing appropriate care to pregnant women with CHDs [13].

Each woman’s residential address as listed on the discharge record and the addresses of all birthing hospitals were geocoded with ArcGIS [14] and the NYS Street and Address Maintenance (SAM) Program [15]. The *gmapsdistance* package in R was used to access the Google Maps Distance Matrix API to calculate drive distance, drive time, and transit time from each identified residential address to the actual delivery hospital and the Level 3/RPC centers nearest to that address [16]. Travel time was estimated between one origin and one destination by calling the Google Distance API iteratively. Google API estimates travel time based on historical and current time-of-day and day-of- week road network data while taking into account the traffic congestion and flow [17]. Then, the closest appropriate care center in terms of driving time was identified. Public transit calculations were limited to only those areas where this information was available through Google API; however, most major public transit systems in NYS are included. The difference in travel times to the closest appropriate care location and delivery hospital attended was calculated by subtracting the one-way travel time to closest Level 3 or RPC hospital from the one-way travel time to the delivery hospital attended. A negative value indicated that the attended hospital was farther away from the residence than the closest appropriate care location, whereas a positive value indicated that the attended hospital was closer to the residence than the closest appropriate care location.

**Fig. 1:**
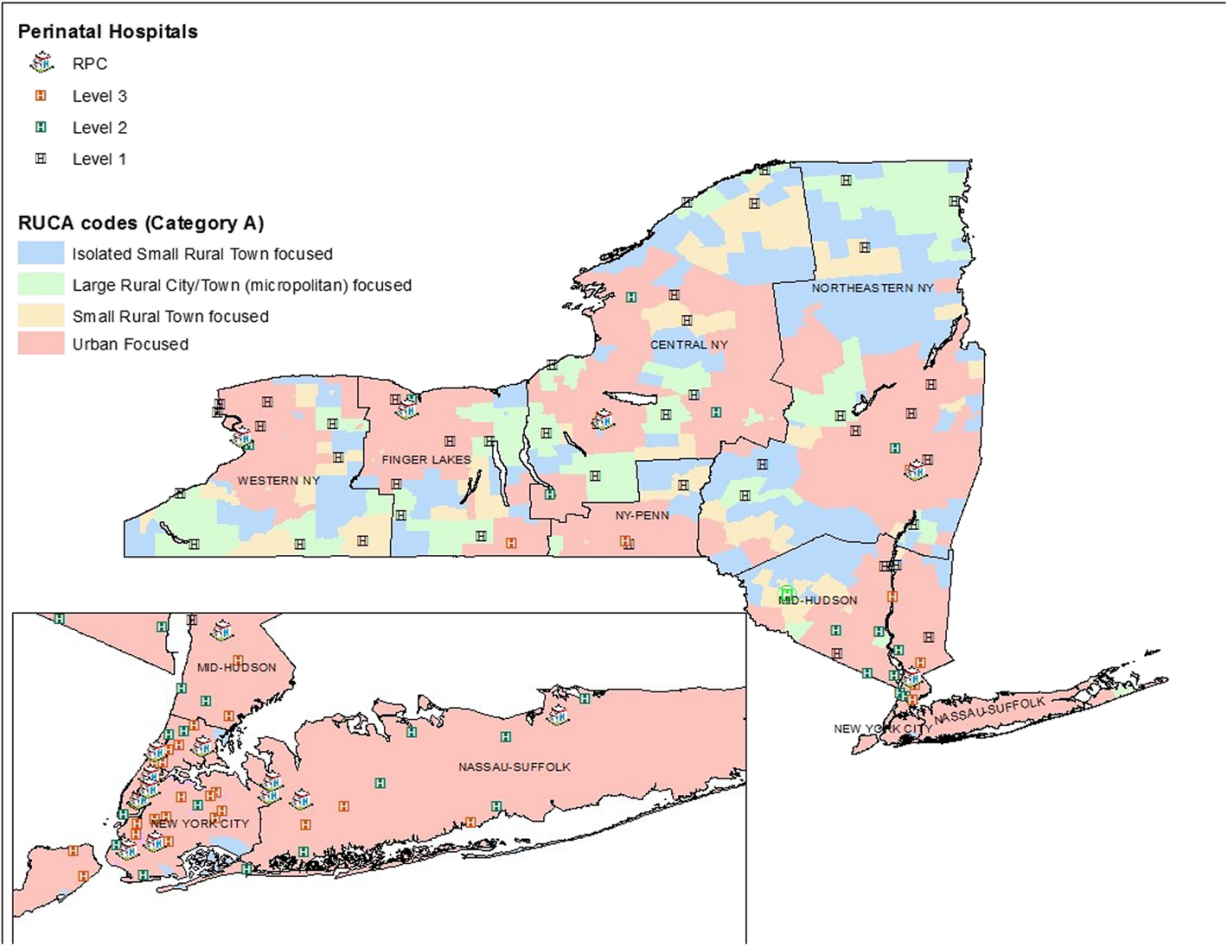
Map showing census tract level RUCA codes and New York State Perinatal Birthing Hospitals by Level and HSA areas

Based on the geocoded residential address, each woman was assigned to one of the eight health service areas (HSAs) in NYS, as shown in Fig. 1 [6]. Women were further classified into one of two regions: New York City (NYC) (HSA 7) or outside of NYC (HSA 1–6 and 8). Analyses by region were conducted due to differences in access to care in extremely urban locations, such as NYC. Each woman’s residence was also classified using the 2010 Rural-Urban Commuting Area (RUCA) Codes, which classifies locations according to census tracts, based on data from the 2010 Census and the 2006–2010 American Community Survey [18]. RUCA Categorization A was used to place women in one of four residential groups: urban, large rural city/town (micropolitan), small rural town, or isolated small rural town. For analyses with small sample sizes, RUCA categorization C was utilized where the three rural categories of categorization A were combined to create a single rural group. Data from the 2010–2014 American Community Survey (ACS-ATSDR) provided neighborhood information at the block group level for each woman on percent of persons above the 200% federal poverty line (FPL) near her residence. Stratification at 200% FPL is in concert with published definitions of low-income families and additionally identifies families who may be eligible for governmental supports [19].

Bivariate analyses were conducted to assess differences in characteristics of pregnant women with CHDs by region and travel patterns. Linear regression was used to evaluate predictors of one-way drive and public transit time to appropriate care for pregnant women with CHDs. Predictors considered included neighborhood (urban/rural, percent of persons above the 200% poverty line) and patient level characteristics (CHD severity, age, race, insurance status). Logistic regression was used to examine predictors of receiving care at a level 3 or higher birthing facility. Along with the sociodemographic and patient level characteristics, drive/transit time to the closest level 3 or RPC were also included in the model.

For all analyses, statistical significance was assigned based on a *p*-value of < 0.05. Model fit was evaluated using deviance and Pearson statistics tests. All statistical analyses were conducted using SAS 9.4, R 3.3.0, and ArcGIS 10.4.1.

## Results

Within the NYSDOH regionalized perinatal services classification system during this time period, there were 49 Level 1 hospitals, 26 Level 2 hospitals, 34 Level 3 hospitals, and 17 RPCs (Fig. 1). Within the five counties that compose the NYC region, there are no Level 1 hospitals, but the region possesses 65% (*N* = 22) of the Level 3 hospitals and 53% (*N* = 9) of the RPCs in the state. Outside of the NYC region, Level 1 and Level 2 hospitals are more common than Level 3 and RPC hospitals. In the area surrounding NYC, in particular HSAs 6 (Mid-Hudson) and 8 (Nassau-Suffolk), the number of Level 1 and 2 hospitals outnumber the number of Level 3 and RPC hospitals, but these areas still contain more high-level facilities than other HSAs in Upstate NY. Upstate NY HSAs 1–5 each contain one RPC and one Level 3 hospital, except for the smaller HSA 4 (NY-PENN), which does not contain an RPC. All but one of the RPCs in Upstate NY are also pediatric cardiac surgery centers, allowing for specialized cardiac care if needed. Furthermore, all of the Level 3 and RPC hospitals in the state are located in urban areas, whereas Level 1 and 2 hospitals were located in both urban and rural locations.

Between 2008 and 2013, there were 922 delivery hospitalizations of NYS residents of childbearing age (15–44 years old) with CHDs in the SPARCS data set. After exclusion of non-geocoded addresses, 909 women remained for analysis, with about 42% residing in one of the five NYC counties. Parturient women in NYC were significantly more likely to have severe CHDs and were significantly more likely to have coexisting heart failure than women outside NYC (Table 1). A vast majority of parturient women with CHD in NYC delivered at a high-level facility (91.88%) as compared to only 62.24% in areas outside NYC. Women in NYC were also more likely to deliver at hospitals that were designated teaching facilities and had significantly higher charges than women outside NYC. Reflecting the population distribution across New York State, parturient women with CHDs outside of NYC were more likely to be white, non-Hispanic. There were no other significant differences in cardiac or other comorbidities, age, insurance type, or delivery method between the two regions.

**Table 1:** Characteristics of Pregnant Women in NY with CHD Admitted for Delivery, by Region, SPARCS, 2008–2013

	NYS (Total = 909) N(%)	NYC (Total = 382) N(%)	Outside NYC (Total = 527) N(%)	***P*** value
**PATIENT CHARACTERISTICS**				
**CHD Severity**				0.0042
Severe	46 (5.06)	28 (7.33)	18 (3.42)	
Mild-Moderate	617 (67.88)	266 (69.63)	351 (66.60)	
Isolated Atrial Septal Defect	246 (27.06)	88 (23.04)	158 (29.98)	
**Cardiovascular Comorbidities**	253 (27.83)	109 (28.53)	144 (27.32)	0.69
Hypertension	12 (1.32)	5 (1.31)	7 (1.33)	0.62
Hyperlipidemia	3 (0.33)	2 (0.52)	1 (0.19)	NC
Coronary Artery Disease	5 (0.55)	1 (0.26)	4 (0.76)	NC
**Other Cardiovascular**	178 (19.58)	71 (18.59)	107 (20.30)	0.52
Conduction Disorder	72 (7.92)	34 (8.90)	38 (7.21)	0.35
Venous Disorder/Phlebitis	8 (0.88)	5 (1.31)	3 (0.57)	0.24
Heart Failure	19 (2.09)	14 (3.66)	5 (0.95)	0.0047
**Other Comorbidities**				
Diabetes Mellitus	17 (1.87)	11 (2.88)	6 (1.14)	0.06
Other Endocrine, Non-Diabetes Mellitus	58 (6.38)	19 (4.97)	39 (7.40)	0.14
Birth Defects (Non-CHD)	26 (2.86%)	10 (2.62)	16 (3.04)	0.71
Musculoskeletal	33 (3.63)	17 (4.45)	16 (3.04)	0.26
**Respiratory/Pulmonary**	119 (13.09)	57 (14.92)	62 (11.76)	0.16
Injury/Trauma	5 (0.55)	3 (0.79)	2 (0.38)	NC
Neurological/Central Nervous System	20 (2.20)	11 (2.88)	9 (1.71)	0.23
Immunology/Rheumatology/Allergy	11 (1.21)	5 (1.31)	6 (1.14)	0.82
Infectious Disease	62 (6.82)	27 (7.07)	35 (6.64)	0.80
Mental Health	96 (10.56)	26 (6.81)	70 (13.28)	0.0017
Hematologic	148 (16.28)	72 (18.85)	76 (14.42)	0.07
Neoplasms	30 (3.30)	17 (4.45)	13 (2.47)	0.10
**Gastrointestinal**	43 (4.73)	23 (6.02)	20 (3.80)	0.12
Renal and Other Genitourinary	8 (0.88)	2 (0.52)	6 (1.14)	NC
Genitourinary/Gynecological	50 (5.50)	25 (6.54)	25 (4.74)	0.24
**Number of Other Comorbidities**				
0	421 (46.31)	164 (42.93)	257 (48.77)	0.22
1–2	418 (45.98)	187 (48.95)	231 (43.83)	
3+	70 (7.70)	31 (8.12)	39 (7.40)	
**Age, Continuous**	30.54 + 6.21	30.50 + 6.20	30.58 + 6.23	0.84
**Age, Categorical**				0.17
15–19 years	25 (2.75)	7 (1.83)	18 (3.42)	
20–24 years	161 (17.71)	77 (20.16)	84 (15.94)	
25–29 years	212 (23.32)	78 (20.42)	134 (25.43)	
30–34 years	256 (28.16)	111 (29.06)	145 (27.51)	
35–39 years	174 (19.14)	78 (20.42)	96 (18.22)	
40–44 years	81 (8.91)	31 (8.12)	50 (9.49)	
**Race/Ethnicity**				< 0.0001
Hispanic	150 (16.50)	105 (27.49)	45 (8.54)	
Non-Hispanic White	518 (56.99)	134 (35.08)	384 (72.87)	
Non-Hispanic Black	106 (11.66)	65 (17.02)	41 (7.78)	
Non-Hispanic Other	135 (14.85)	78 (20.42)	57 (10.82)	
**Insurance**				0.57
Private	730 (80.31)	313 (81.94)	417 (79.13)	
Federal	142 (15.62)	63 (16.49)	101 (19.17)	
Self Pay	15 (1.65)	6 (1.57)	9 (1.70)	
**NEIGHBORHOOD CHARACTERISTICS**				
**RUCA Category**				NC
NYC	382 (42.02)	382 (100)	0 (0)	
Non-NYC Urban	496 (54.57)	0 (0)	496 (94.12)	
Non-NYC Rural	31 (3.41)	0 (0)	31 (5.88)	
**Percent Above 200% Poverty Line**				< 0.001
0–25%	79 (8.69)	46 (12.04)	33 (6.26)	
26–50%	199 (21.89)	130 (34.03)	69 (13.09)	
51–75%	265 (29.15)	99 (25.92)	166 (31.50)	
76–100%	366 (40.26)	107 (28.01)	259 (49.15)	
**CHARACTERISTICS OF HOSPITAL ATTENDED**				
**Closest Level 3/RPC Hospital**				0.01
Yes	192 (21.12)	65 (17.02)	127 (24.10)	
No	717 (78, 88)	317 (82.98)	400 (75.90)	
**Teaching Status**				< 0.0001
Teaching	807 (88.78)	370 (96.86)	437 (82.92)	
Non-Teaching	102 (11.22)	12 (3.14)	90 (17.08)	
** Hospital Level**				NC
Level 1	87 (9.57)	0 (0)	87 (16.51)	
Level 2	137 (15.07)	27 (7.07)	110 (20.87)	
Level 3	179 (19.69)	110 (28.80)	69 (13.09)	
Level 4 (RPC)	500 (55.01)	241 (63.08)	259 (49.15)	
Not classified	6 (0.66)	4 (1.05)	2 (0.38)	
**CHARACTERISTICS OF DELIVERY**				
**Delivery Method**				0.11
Vaginal Delivery	512 (56.33)	227 (59.43)	285 (54.08)	
Operative Vaginal Delivery	12 (1.32)	7 (1.83)	5 (0.95)	
Cesarean Delivery	385 (42.35)	148 (38.74)	237 (44.97)	
**Length of Stay (median, IQR)**	3.00 (2.00–4.00)	3.00 (2.00–4.00)	3.00 (2.00–4.00)	0.0486
**Total Charges ($)(median, IQR)**	13,764.36 (8391.00-21,007.07)	16,823.65 (11,423.25-25,402.68)	11,371.55 (7108.50-18,349.97)	< 0.0001

*A high- level birthing facility is defined as a Regional Perinatal Center (Level 4) or a Level 3 birthing hospital in New York State

**For variables with a cell size less than 5, *p*-values were not calculated (NC)

### Drive time

Parturient women with CHDs in NYS traveled a median one-way drive time of 19.7 min (Range:1.0–100.5 min; Table 2) to their listed delivery hospital. Women in the Finger Lakes and NYC had the shortest median drive times of 17.6 and 17.8 min, respectively. Women in NY-Penn (24.5 min), Nassau-Suffolk (24.1 min), and Mid-Hudson (23.9 min) health service areas had the longest median drive times (Additional file 1 Table A1).

**Table 2:** One-Way Drive Times and One-Way Transit Times From CHD Case Residential Address to the Actual Delivery Hospital, Closest Birthing Hospital and Closest Level 3/RPC Birth Center, Stratified by Health Service Area

		One-way drive time between residential address and	
		Actual Delivery Hospital	Closest Level 3/RPC Birthing Hospital
	**Total addresses (n)**	**Median (Range), minutes**	**Median (Range), minutes**
**All New York State**	909	19.7 (1.0–100.5)	14.0 (1.3, 198.0)
Western NY	92	19.1 (1.9–100.5)	20.9 (1.9, 109.1)
Finger Lakes	58	17.6 (2.9–96.2)	17.7 (2.1, 67.1)
Central NY	60	21.4 (4.1–84.1)	24.3 (4.1, 127.4)
NY-Penn	7	24.5 (13.4–43.8)	25.3 (10.7, 57.8)
Northeastern NY	77	19.72 (1.8–95.0)	36.2 (4.8, 198.0)
Mid-Hudson	91	23.9 (4.0–87.5)	25.3 (3.6, 73.4)
NYC	374	17.8 (1.8–55.6)	9.5 (1.8, 32.4)
Nassau-Suffolk	150	24.1 (1.0–69.7)	15.6 (1.3, 62.4)
		**One-way public transit time between residential address and**	
		**Actual Delivery Hospital**	**Closest Level 3/RPC Birthing Hospital**
	**Addresses with public transit available (%)**	**Median (Range), minutes**	**Median (Range), minutes**
**All New York State**	722 (79.4)	45.8 (1.9–411.2)	27.6 (3.1, 412.7)
Western NY	72 (78.3)	69.2 (5.1–238.1)	56.5 (4.7, 129.5)
Finger Lakes	43 (74.1)	49.8 (7.5–411.2)	56.9 (17.4, 214.7)
Central NY	39 (65.0)	62.0 (11.2–293.3)	60.1 (12.5, 243.1)
NY-Penn	2 (28.6)	40.7 (33.3–48.2)	40.7 (33.3, 48.2)
Northeastern NY	36 (46.8)	52.4 (7.8–232.5)	80.3 (11.8, 172.0)
Mid-Hudson	53 (58.2)	76.1 (15.2–299.4)	44.2 (10.3, 232.4)
NYC	373 (99.7)	36.5 (4.3–149.1)	16.1 (3.1, 62.5)
Nassau-Suffolk	106 (70.7)	78.2 (1.9–204.4)	60.2 (3.9, 224.6)

Across the whole state, the median one-way drive time to the closest Level 3 or RPC was 14.0 min (Range: 1.3–198.0 min), a shorter drive time than the listed delivery hospitals. Women in NYC had the shortest median drive time to appropriate care at 9.5 min, whereas women in Northeastern NY had the longest median drive time at 36.2 min.

### Transit time

The median one-way public transit time for parturient women with CHD in NYS to their listed delivery hospital was 45.8 min (Range: 1.9–411.2 min; Table 2). Women with CHD in NYC had the shortest median transit time (36.5 min) to their delivery hospital, whereas those in Nassau-Suffolk (78.2 min) and Mid-Hudson (76.1 min) had the longest median transit time.

Across the whole state, the median one-way public transit time to the closest Level 3 or RPC was 27.6 min (Range: 3.1–412.7 min). Women with CHD in NYC had the shortest median transit time to appropriate care at 16.1 min, whereas women with CHD in Northeastern NY had the longest median transit time at 80.3 min.

### Utilization of appropriate care facilities

Approximately 21% of women with CHD attended their closest appropriate care facility for delivery, whereas 18% attended a closer hospital and 61% attended a hospital further away (Table 3). Hispanic and non-Hispanic Black women with CHD were more likely than non-Hispanic Whites to travel farther than their closest appropriate care center (63 and 62% respectively compared to 55% for non-Hispanic Whites). Those who attended a closer, lower level hospital were less likely to have a non-cardiovascular-related comorbidity, had a shorter length of stay, lower total charges, and were less likely to attend a teaching hospital than those who attended their closest appropriate care facility. Half of the women who attended a closer, lower level facility were from Western and Northeastern NY, some of the most rural areas of the state.

**Table 3:** Characteristics of Parturient Women by Travel Pattern to the Nearest High-Level Birthing Hospital, SPARCS, 2008–2013

	Attended the Nearest High Level Birthing Hospital (***N*** = 192) N(%)	Attended a Hospital Closer than the Nearest High Level Birthing Hospital (***N*** = 162) N(%)	***P*** value	Attended a Hospital Farther than the Nearest High Level Birthing Hospital (***N*** = 555) N(%)	***P*** value
**PATIENT CHARACTERISTICS**					
**CHD Severity**			0.41		0.84
Severe	11 (5.73)	5 (3.09)		30 (5.41)	
Mild-Moderate	133 (69.27)	110 (67.90)		374 (67.39)	
Isolated Atrial Septal Defect	48 (25.00)	47 (29.01)		151 (27.21)	
**Cardiovascular-Related Comorbidities**			0.08		0.07
Yes	48 (25.00)	28 (17.28)		177 (31.89)	
No	144 (75.00)	134 (82.72)		378 (68.11)	
**Other Comorbidities**			0.0375		0.66
Yes	109 (56.77)	74 (45.68)		305 (54.95)	
No	83 (43.23)	88 (54.32)		250 (45.05)	
**Age, Continuous**	29.62 + 6.38	30.75 + 6.21	0.09	30.81 + 6.14	0.02
**Age, Categorical**			0.68		0.003
15–19 years	10 (5.21)	6 (3.70)		9 (1.62)	
20–24 years	32 (16.67)	25 (15.43)		104 (18.74)	
25–29 years	60 (31.25)	41 (25.31)		111 (20.00)	
30–34 years	44 (22.92)	41 (25.31)		171 (30.81)	
35–39 years	32 (16.67)	35 (21.60)		107 (19.28)	
40–44 years	14 (7.29)	14 (8.64)		53 (9.55)	
**Race/Ethnicity**			0.005		0.14
Hispanic	37 (19.27)	18 (11.11)		95 (17.12)	
Non-Hispanic White	108 (56.25)	120 (74.07)		290 (52.25)	
Non-Hispanic Black	25 (13.02)	15 (9.26)		66 (11.89)	
Non-Hispanic Other	22 (11.46)	9 (5.56)		104 (18.74)	
**Insurance**			NC		NC
Private	143 (74.48)	120 (74.07)		467 (84.14)	
Federal	45 (23.44)	37 (22.84)		82 (14.77)	
Self -Pay	4 (2.08)	5 (3.09)		6 (1.08)	
**NEIGHBORHOOD CHARACTERISTICS**					
**HSA of Patient Residence**			NC		NC
1-Western NY	14 (7.29)	41 (25.31)		37 (6.67)	
2-Finger Lakes	29 (15.10)	13 (8.02)		16 (2.88)	
3-Central NY	13 (6.77)	15 (9.26)		32 (5.77)	
4-NY-PENN	5 (2.60)	1 (0.62)		1 (0.18)	
5-Northeastern NY	9 (4.69)	39 (24.07)		29 (5.23)	
6-Mid-Hudson	13 (6.77)	27 (16.67)		51 (9.19)	
7- NYC	65 (33.85)	11 (6.79)		298 (53.69)	
8-Nassau-Suffolk	44 (22.92)	15 (9.26)		91 (16.40)	
**RUCA Category**			NC		NC
NYC	65 (33.85)	12 (7.41)		305 (54.95)	
Non-NYC Urban	126 (65.63)	129 (79.63)		241 (43.42)	
Non-NYC Rural	1 (0.52)	21 (12.96)		9 (1.62)	
**Percent Above 200% Poverty Line**					
0–25%	16 (8.33)	11 (6.79)	0.76	52 (9.37)	0.49
26–50%	25 (18.23)	36 (22.22)		128 (23.06)	
51–75%	58 (30.21)	50 (30.86)		157 (28.29)	
76–100%	83 (43.23)	65 (40.12)		218 (39.28)	
**CHARACTERISTICS OF HOSPITAL ATTENDED**					
**Teaching Status**			< 0.0001		0.003
Teaching	17 (91.15)	95 (58.64)		537 (96.76)	
Non-Teaching	17 (8.85)	67 (41.36)		18 (3.24)	
**Hospital Level**			NC		NC
Level 1	0 (0)	65 (40.12)		22 (3.96)	
Level 2	0 (0)	94 (58.02)		43 (7.75)	
Level 3	81 (42.19)	0 (0)		98 (17.66)	
Level 4 (RPC)	111 (57.81)	0 (0)		389 (70.09)	
Not classified	0 (0)	3 (1.85)		3 (0.54)	
**CHARACTERISTICS OF DELIVERY**					
**Delivery Method**			NC		NC
Vaginal Delivery	117 (60.94)	94 (58.02)		301 (54.23)	
Operative Vaginal Delivery	1 (0.52)	2 (1.23)		9 (1.62)	
Cesarean Delivery	74 (38.54)	66 (40.74)		245 (44.14)	
**Length of Stay (median, IQR)**	3 (2–4)	2 (2–3)	0.05	3 (2–4)	0.09
**Total Charges ($) (median, IQR)**	$12,317 ($8348–$17,776)	$7767 ($6000–$12,355)	< 0.0001	$16,480 ($10,257–$25,403)	< 0.0001

*A high level birthing facility is defined as a Regional Perinatal Center (Level 4) or a Level 3 birthing hospital in New York State

**For variables with a cell size less than 5, p-values were not calculated (NC)

Women with CHD who delivered at a hospital farther than their nearest appropriate care facility exhibited no significant difference in disease severity, cardiovascular comorbidities, or other comorbidities than those who attended their closest appropriate care facility (Table 3). Women who traveled further were older, more likely to attend a teaching hospital, and had higher total charges. Additionally, women who traveled farther for delivery than their nearest appropriate care facility still typically attended Level 3 (18%) and RPC (70%) facilities. The majority of these women were from the HSAs surrounding NYC in the Mid-Hudson (21.67%) and Nassau-Suffolk (41.67%) areas. There were 403 women whose closest Level 3 or RPC facility was a Level 3. Among these women, *N* = 290 (71.96%) bypassed their Level 3 to go to a RPC while *N* = 73 (18.11%) bypassed their Level 3 to go to a Level 3 that was further away (Additional file 1 Table A2).

Finally, 12% of women who traveled farther than the closest appropriate care hospital attended a lower level facility. Half of these women lived in the HSAs of Western NY (37.21%) and the Finger Lakes (16.28%). Pregnant women from the Nassau-Suffolk area (30.23%) also accounted for a large percentage of women who attended a lower level facility that was farther away.

### Predictors of Drive & Transit Times

In unadjusted models, race/ethnicity, insurance, percent of the population above 200% poverty line, and rurality were significant predictors of drive time. Increasing age was associated with shorter drive time. Those who lived in areas with less than 50% of the population above 200% of the poverty line or women who were of a race/ethnicity other than non-Hispanic Whites had significantly lower drive and/or transit time. Those in rural areas or those who had federal insurance or were uninsured had higher drive times. On multivariate adjustment, severity of defect, race/ethnicity and rurality remained significant predictors of one-way drive and transit times to the nearest appropriate care facility (Table 4). Non-Hispanic Blacks had the shortest drive (6.7 min less than non-Hispanic Whites) and public transit time (11.7 min less than non-Hispanic Whites) of all races. Hispanics had a 4 min shorter drive time than non-Hispanic Whites. Drive time and transit time for non-NYC rural residents were 73.97 and 87.36 min more than NYC residents. Model Fit statistics for the multivariate models are presented in Additional file 1 (Figs. A1 and A2).

**Table 4:** Results from the Linear Model to Examine Patient and Neighborhood Variables and One-Way Drive Time and One-Way Transit Time to Nearest Level 3 or RPC Birthing Hospital (n = 909), New York, 2008–2013

	One-way Drive Time, minutes		One-way Transit Time, minutes	
	Unadjusted β (95% CI)	Adjusted β (95% CI)	Unadjusted β (95% CI)	Adjusted β (95% CI)
**Intercept**		13.58 (6.96, 20.20)		26.48 (6.03, 46.93)
**Age (years)**	−0.22 (−0.43,0.00)	−0.11 (− 0.28, 0.06)*	0.26 (− 0.31, 0.82)	− 0.11 (− 0.63, 0.42)
**CHD Severity**				
Non-Severe (Isolated Atrial Septal Defect)		Reference		Reference
Severe (Severe/Mild Moderate)	1.91 (− 1.09,4.91)	2.37 (0.18, 4.55)*	−5.34 (− 13.14, 2.46)	1.71 (− 4.90, 8.32)
**Cardiovascular Comorbidities**				
Yes	−1.08 (− 4.05,1.90)	1.22 (−0.94, 3.38)	− 1.48 (−9.39,6.43)	1.54 (− 5.11, 8.19)
No		Reference		Reference
**Other Comorbidities**				
Yes	−1.81 (−4.48,0.86)	−0.85 (−2.79, 1.09)	−3.99 (− 11.09, 3.10)	−1.48 (−7.42, 4.46)
No		Reference		Reference
**Race**				
Hispanic	−10.71 (− 14.33, −7.10)*	−4.08 (− 7.11, − 1.04)*	−24.72 (− 33.80,-15.65)*	−4.13 (− 12.89, 4.63)
Non-Hispanic White		Reference		Reference
Non-Hispanic Black or African American	−12.24(− 16.40,-8.09)*	−6.73 (− 10.04, − 3.14)*	−27.20 (− 37.55, − 16.85)*	−11.69 (− 21.21, − 2.16)*
Non-Hispanic Other Race	−7.15 (− 10.92,-3.38)*	−1.66 (− 4.56, 1.25)	− 14.61 (− 24.34, − 4.89)*	0.78 (− 7.85, 9.41)
**Insurance**				
Private		Reference		Reference
Federal	4.41 (0.96, 7.86)*	0.73 (−1.89, 3.35)	−2.52 (− 11.75, 6.70)	−3.62 (− 11.69, 4.45)
Self Pay	15.90 (5.49, 26.31)*	5.43 (− 2.17, 13.03)	− 7.98 (− 38.22, 22.27)	0.05 (− 25.11, 25.20)
**Percent of Persons Above 200% Poverty Line**				
0–25%	−2.04 (− 7.01, 2.92)	2.59 (− 1.25, 6.44)	− 22.85 (− 35.45, − 10.26)*	−5.58 (− 17.28, 6.12)
26–50%	− 4.52 (− 8.04, − 0.99)*	0.25 (− 2.68, 3.17)	− 23.08 (− 32.01, − 14.15)*	−3.54 (− 12.33, 5.24)
51–75%	1.66 (−1.56, 4.89)	1.24 (−1.18, 3.67)	−3.00 (− 11.59,5.59)	2.99 (− 4.54, 10.52)
76–100%		Reference		Reference
**Rurality**				
NYC		Reference		Reference
Non-NYC Urban	13.97 (11.99, 15.95)*	12.73 (10.55, 14.90)*	54.35 (48.51, 60.19)*	52.13 (45.79, 58.47)*
Non-NYC Rural	76.37 (70.95, 81.79)*	73.97 (68.40, 79.54)*	82.29 (36.98, 127.60)*	87.36 (41.46, 133.27)*

*P value < 0.05

### Predictors of utilization of Care at an Appropriate Facility

Finally, we assessed the predictors of receiving care at an appropriate facility using a logistic regression model. In the unadjusted models, those with cardiovascular or other comorbidities and racial or ethnic minorities were more likely to receive care at a Level 3 or RPC. Increasing drive time to a higher-level care center, federal insurance and non-NYC residence were associated with lower odds of attending an appropriate care center. After multivariate adjustment, women with cardiovascular morbidities and other comorbidities had significantly greater odds of attending an appropriate care hospital. For each one-minute increase in drive time to the closest appropriate care facility, there was a 4% decrease in the odds of delivery at an appropriate care facility. Women in areas with moderate levels of income were less likely to deliver at high level hospital than those residing in neighborhoods with lowest or highest levels of incomes. Although Hispanic and Non-Hispanic Black women lived significantly closer to high-level facilities (Table 4), their odds of actual delivery at a high-level center were not significantly different than those for non-Hispanic White women on multivariate adjustment (Table 5). After adjustment for drive time and other factors, non-NYC urban women were significantly less likely to deliver at an appropriate care center as compared to rural and NYC residents. Results were similar in multivariate models with transit time (data not shown). Model fit statistics are presented in Additional file 1 (Table A3).

**Table 5:** Results from the Logistic Model to Examine Predictors of Receiving Care at a Level 3 or RPC Birthing Hospital (*n* = 909), New York, 2008–2013

	Unadjusted OR (95% CI)	Adjusted OR (95% CI)
**Drive Time to Closest Level 3 or RPC, minutes**	0.95 (0.94, 0.96)*	0.96 (0.94, 0.97)*
**Age (years)**	1.00 (0.97, 1.02)	0.96 (0.93, 0.99)*
**CHD Severity**		
Non-Severe (Isolated Atrial Septal Defect)	Reference	Reference
Severe (Severe/Mild Moderate)	1.25 (0.90, 1.74)	1.38 (0.94, 2.02)
**Cardiovascular Comorbidities**		
Yes	1.95 (1.35, 2.82)*	2.29 (1.49, 3.52)*
No	Reference	Reference
**Other Comorbidities**		
Yes	1.72 (1.27, 2.33)*	1.86 (1.31, 2.65)*
No	Reference	Reference
**Race**		
Hispanic	2.09 (1.33, 3.28)*	0.94 (0.53, 1.68)
Non-Hispanic White	Reference	Reference
Non-Hispanic Black or African American	2.70 (1.54, 4.74)*	1.35 (0.69, 2.66)
Non-Hispanic Other Race	3.12 (1.84, 5.30)*	2.24 (1.22, 4.10)*
**Insurance**		
Private	Reference	Reference
Federal	0.67 (0.46, 0.97)*	0.70 (0.44, 1.12)
Self Pay	0.62 (0.21, 1.84)	0.67 (0.15, 2.88)
**Percent of Persons Above 200% Poverty Line**		
0–25%	1.54 (0.82, 2.87)	1.10 (0.50, 2.41)
26–50%	0.96 (0.65, 1.43)	0.34 (0.20, 0.60)*
51–75%	0.85 (0.60, 1.22)	0.76 (0.50, 1.18)
76–100%	Reference	Reference
**Rurality**		
NYC	Reference	Reference
Non-NYC Urban	0.16 (0.11, 0.24)*	0.24 (0.15, 0.40)*
Non-NYC Rural	0.04 (0.02, 0.09)*	0.57 (0.17, 1.89)

*P value < 0.05

## Discussion

The majority of parturient women with CHDs in New York State appear to meet the American College of Cardiology and the American Heart Association recommendations for delivery at facilities with appropriate delivery care, as 75% of CHD patients delivered at Level 3 and RPC hospitals [6]. Moreover, women with more serious defects typically attended high level facilities, with 85% of severe CHD patients and 75% of mild-to-moderate CHD patients meeting the recommendations. The present results mirror the findings by Fernandes et al. (2015) and Maxwell et al. (2014) [20, 21], who reported that approximately 25% of CHD patients attended non-specialty hospitals for surgical care, indicating similar utilization choices for different types of medical needs among CHD patients. With 51 high-level birthing facilities in NYS, the travel time to the closest appropriate care facility, whether by personal or public transport, is relatively short for most women with CHD, especially those in the NYC HSA. Since most Level 3 and RPC centers are located in urban and metropolitan areas, we expected women residing in rural locations to be the farthest from high-level facilities. Still, only 6% of pregnant women with CHDs resided in areas classified as rural and this subgroup with poor access to care represents a very small portion of the CHD population.

Parturient women with CHD in the northernmost HSA areas of Northeastern and Central NY lived relatively close to a Level 1 or 2 birthing hospital but must travel in excess of 100 miles in some instances to reach the nearest Level 3 or RPC facility. Moreover, the logistic regression model showed that increases in drive times resulted in a decreased likelihood that women received care at a Level 3 or RPC facility. Therefore, women in Western and Northeastern NY and those in rural towns across the state were more likely to deliver at a hospital closer than their nearest high-level birthing facility, similar to the findings of a recent study of parturient women residing in rural and remote towns [22]. Consequently, primary care providers should include discussions of delivery locations with women early in pregnancy to determine their care needs and how far they would need to travel for delivery at an appropriate care center. After adjustment for time to travel, CHD severity, other comorbidities, socioeconomic and other demographic factors, rural women were as likely to deliver at higher level birthing facility as their counterparts in NYC. This suggests that most rural urban differences can be explained by geographic and sociodemographic barriers to access to care among rural women. However, after multivariate adjustment, women in non-NYC urban areas are at a distinct disadvantage with regards to utilizing appropriate delivery care suggesting that factors in addition to geographic proximity determine where women residing in these areas deliver their babies.

Non-Hispanic Black and Hispanic women had a significantly shorter drive time to a high-level birthing facility. This is not surprising, as despite recent declines in residential racial segregation, Blacks and Hispanics are more likely to live in urban inner-city areas that have higher numbers of health care facilities [23]. Despite being closer to an appropriate center than other race/ethnicities, Black and Hispanic women were not more likely to utilize services based on multivariate models. In addition, Black and Hispanic women were also more likely to travel farther than their closest appropriate center for delivery which suggest that factors other than geographic proximity may play a role in determining where delivery occurs in these women. Previous research has shown that despite the advantage of residence in proximity to higher level centers, these minorities in particular have a higher prevalence of adverse birth outcomes [24]. Even in urban areas with a high density of delivery hospitals, Black patients often receive care at poorer quality hospitals resulting in adverse infant outcomes [25]. Future research in this area should be directed to examining racial-ethnic disparities in maternal and fetal health among women with CHD despite proximity to high level delivery care. Similarly, poor or mixed income neighborhoods had significantly shorter transit times but that did not translate into an increased utilization of high-level delivery services for women in these areas. Rather women in mixed income neighborhoods were almost half as likely to utilize high-level delivery services than women from high income neighborhoods. These results are consistent with research on health care utilization that show that racial and socioeconomically disadvantaged groups in need of specialized services such as adjuvant chemotherapy in breast cancer patients or radiation therapy among rectal cancer patients are less likely to receive appropriate services despite adequate geographic access [26, 27].

Most women in NYC attend a high-level hospital because it is the only type of facility within their health service area. Furthermore, most RPCs in NYC are also pediatric cardiac surgery centers or have 1 a short distance away. While the average distance to appropriate care for NYC residents is approximately one mile, some parturient women with CHDs traveled a farther distance to attend another facility, which also provided appropriate care. These women may have attended a facility farther away due to the regionalization of care, their physician’s hospital affiliations, insurance network, or their personal preferences, as previously discussed by Fernandes et al. (2015) [20].

Women in the HSAs surrounding NYC, including the Mid-Hudson and Nassau-Suffolk HSAs, were also more likely to travel farther than their nearest high-level facility for delivery. Previous examinations of travel patterns for CHD patients undergoing surgery showed that 51% of CHD patients who attended a specialty care center traveled farther than the nearest one [28]. While women in both of these areas have a number of appropriate care facilities within close proximity, 41% of women in the Mid-Hudson area who traveled farther to another appropriate care facility delivered at a NYC hospital, compared to only 15% of women in the Nassau-Suffolk area, demonstrating different travel patterns surrounding NYC.

Interestingly, women from the Nassau-Suffolk area also accounted for nearly a third of women who attended a Level 1 or 2 hospital that was farther away than the closest appropriate care facility. Women in western NYS were also more likely to attend a lower level hospital that was farther away. Although women traveled further for lower level care, the additional distance traveled was relatively small. The present study was unable to assess characteristics of women who attended a lower level hospital located farther away than the closest appropriate care facility due to small sample sizes; however, additional analyses are warranted to understand the behavior of this subpopulation.

Based on the Anderson behavioral model, there are components to the use of health services: predisposing characteristics, enabling resources, and need [29]. We included two enabling factors (geographic proximity to appropriate care facilities and availability of public transportation) and multiple predisposing characteristics, such as age, CHD severity, comorbidities, race, and insurance, in our analyses. However, there are other enabling factors that we were unable to examine on an individual level and had to approximate at the census-tract level, such as poverty. Future analyses should seek to examine additional individual-level enabling factors affecting access to appropriate care and healthcare utilization among pregnant women with CHD.

As expected, travel times via public transportation were longer than travel times via motor vehicle. For most of the state, including the well-connected New York City metro area, it would take almost two to three times longer to reach both the actual listed delivery hospital and closest appropriate care facility via public transportation. Therefore, spatial accessibility within urban areas can still pose a challenge, especially for minorities and low-income urban residents who are more likely to depend on public transportation. This compounds the barriers already faced by these individuals regarding accessing appropriate care. Public health leaders should seek to work with public transportation leaders to improve the number of routes that stop at health care facilities to improve accessibility and reduce travel times.

The present study is one of the few studies that has sought to estimate distance to delivery care via both personal and public transportation for women with CHDs, a subgroup of pregnant women that is in greater need of specialty care. In addition, we have examined whether women attended their nearest delivery hospital or traveled further to a hospital with more services, important distinctions in the examination of healthcare utilization. We have also identified areas within NYS that could benefit from additional locations with higher levels of perinatal and maternal care in order to reduce the travel burden for women with CHDs.

A strength of the present study was the high geocoding rate accomplished, with over 98% of the maternal addresses successfully geocoded. Only 13 residents (1.4%) could not be geocoded due to the P.O. Box listed as their address on the medical record. However, the exclusion of these women from the analysis may have resulted in an underestimation of the distance to care since they likely resided in more rural areas farther from care centers.

There are several potential limitations of this study. Due to the different demographic and geographical characteristics of NYS compared to other states, this study may not be generalizable to the entire US population. Moreover, hospitalization records do not contain information on socioeconomic variables, such as income, occupation, and educational level, which are important influencers on proximity and access to care. As a result, the present study relied upon aggregate data at the census tract level in our predictive models on high distance to appropriate care. In some instances, the census data assigned to an individual based on her residence may not be reflective of her actual status. We did not have access to birth records linkages which may provide a record of maternal characteristics. The small sample size limited our analysis of demographic characteristics, especially within analyses of travel patterns. The small sample did not allow for analyses by severity of CHD and as only 32 women (4%) in the sample resided in rural areas, it was difficult to assess the association between rural location and access to appropriate care. We have combined Level 3 and RPCs to represent adequate level of care in parturient women with CHD. While the main focus of the hierarchal system may be care for high risk newborns, facilities capable of providing specialized care for newborns (Level 3 and RPC) generally include maternal-fetal medicine specialists and comprehensive maternal care as well. If women bypassed a Level 3 center to deliver at an RPC farther away this would result in a negative value for the difference variable between appropriate care and care received. Although RPCs provide a higher level of care, based on the NYS regional perinatal designation Level 3 centers would be determined as the closest appropriate center.

Furthermore, the present study only contained data on deliveries within NYS and women may be able to cross state lines to attend closer facilities that would provide appropriate care; however, we were unable to assess whether and how often this occurs as address information for appropriate care facilities outside of NYS were not available.

## Conclusion

This study characterized proximity to appropriate delivery care for parturient women with CHDs, an important subgroup of CHD patients requiring additional health care utilization. Proximity to adequate care is important for healthy birth outcomes for both the mother and the child, and public health professionals must determine barriers in access to care in order to improve the resources available. Due to the greater travel distances both via personal and public transportation, many CHD patients in rural areas elect to deliver at closer, lower level hospitals, despite the facility’s lack of resources. Our research also suggests that though racial/ethnic and socioeconomically disadvantaged groups may live closer to appropriate care centers, the increased proximity does not always result in improved access for these women. However, despite these challenges, most pregnant women with CHDs in NYS are delivering at high- level facilities and reside relatively short distances from appropriate care, indicating an effective regionalization of appropriate delivery care services. Additional work is still needed to reduce the barriers to appropriate care for women residing in rural areas and women in socioeconomically disadvantaged groups.

## Supplementary information

**Additional file 1: Appendix Tables.** Provides additional analyses on drive and transit times to actual delivery hospital and closest appropriate care facility. Also provides descriptive statistics for level of hospital attended in women travelling further than their closest level 3/RPC (Overall and by region). Additionally, model fit diagnostics are reported.

**Additional file 2: Supplementary Table 1.** Mutually exclusive CHD severity categories with ICD-9-CM codes. A five category CHD ICD-9-CM code classification scheme developed for the surveillance project.

## Data Availability

The data that support the findings of this study are available from the New York State Department of Health SPARCS), but restrictions apply to the availability for of these data, which were used under an agreement for the current study and so are not publicly available.

## Abbreviations

ACS: American Community Survey
ASD: Atrial septal defect
CCS: Clinical Classifications Software
CHD: Congenital heart defect
DRG: Diagnosis-related group
FPL: Federal poverty line
GIS: Geographic Information Systems
HSA: Health Service Area
NY: New York
NYC: New York City
NYS: New York State
NYSDOH: New York State Department of Health
PFO: Patent foramen ovale
RPC: Regional Perinatal Center
RUCA: Rural-Urban Commuting Area
SAM Program: Street and Address Maintenance Program
SPARCS: Statewide Planning and Research Cooperative System
