# Supplementary material for: Proximity to risk-appropriate perinatal hospitals for pregnant women with congenital heart defects in New York state
Source: BMC Pregnancy Childbirth. 2020 Jun 1;20:338. doi: 10.1186/s12884-020-03025-4 (PMC7268637; doi:10.1186/s12884-020-03025-4)
Supplement: Supplementary file 1 — Additional file 1: Appendix Tables. Provides additional analyses on drive and transit times to actual delivery hospital and closest appropriate care facility. Also provides descriptive statistics for level of hospital attended in women travelling further than their closest level 3/RPC (Overall and by region). Additionally, model fit diagnostics are reported. [file 12884_2020_3025_MOESM1_ESM.docx]

Appendix:

**Appendix Table A1. Average One-Way Drive Times and One-Way Transit Times From CHD Case Residential Address to the Actual Delivery Hospital, Closest Birthing Hospital and Closest Level 3/RPC Birth Center, Stratified by Health Service Area**

|  |  | **One-way drive time between residential address and** | |
| --- | --- | --- | --- |
|  |  | **Actual Delivery Hospital** | **Closest Level 3/RPC Birthing Hospital** |
|  | Total addresses (n) | Mean (SD), minutes | Mean (SD), minutes |
| All New York State | 909 | 22.60 (14.85) | 20.43 (20.48) |
| Western NY | 92 | 20.59 (13.93) | 25.60 (20.88) |
| Finger Lakes | 58 | 21.11 (18.36) | 21.70 (13.86) |
| Central NY | 60 | 26.14 (18.87) | 35.74 (28.33) |
| NY-Penn | 7 | 26.11 (11.23) | 29.82 (16.14) |
| Northeastern NY | 77 | 24.68 (19.11) | 47.46 (39.87) |
| Mid-Hudson | 91 | 28.88 (19.55) | 26.29 (14.95) |
| NYC | 374 | 19.43 (10.86) | 10.15 (4.93) |
| Nassau-Suffolk | 150 | 25.82 (12.93) | 18.37 (9.81) |
|  |  | **One-way public transit time between residential address and** | |
|  |  | **Actual Delivery Hospital** | **Closest Level 3/RPC Birthing Hospital** |
|  | Addresses with public transit available (%) | Mean (SD), minutes | Mean (SD), minutes |
| All New York State | 722 (79.4) | 57.14 (46.57) | 45.23 (48.25) |
| Western NY | 72 (78.3) | 66.73 (42.07) | 60.02 (33.00) |
| Finger Lakes | 43 (74.1) | 78.39 (91.89) | 91.91 (102.16) |
| Central NY | 39 (65.0) | 78.69 (60.88) | 79.27 (60.10) |
| NY-Penn | 2 (28.6) | 40.73 (10.56) | 40.73 (10.56) |
| Northeastern NY | 36 (46.8) | 63.28 (52.10) | 80.74 (32.17) |
| Mid-Hudson | 53 (58.2) | 87.39 (51.37) | 69.52 (53.56) |
| NYC | 373 (99.7) | 39.79 (2357) | 19.17 (11.06) |
| Nassau-Suffolk | 106 (70.7) | 85.12 (47.90) | 71.59 (47.70) |

**Appendix Table A2 Descriptive statistics for level of hospital attended in women travelling further than their closest level 3/RPC (Overall and by region)**

| **Level of Closest Level 3 or RPC Hospital** | **Attended Level 1** | **Attended Level 2** | **Attended Level 3** | **Attended RPC** | **Attended Unclassified** |
| --- | --- | --- | --- | --- | --- |
| All NYS |  |  |  |  |  |
| Level 3 | 13 (3.2) | 24 (6.0) | 73 (18.0) | 290 (72.0) | 3 (0.8) |
| RPC | 9 (5.9) | 19 (12.5) | 25 (16.5) | 99 (65.1) | 0 (0) |
| NYC |  |  |  |  |  |
| Level 3 | 0 (0) | 11 (4.9) | 62 (27.6) | 149 (66.2) | 3 (1.3) |
| RPC | 0 (0) | 5 (6.2) | 15 (18.8) | 60 (75.0) | 0 (0) |
| Non-NYC |  |  |  |  |  |
| Level 3 | 13 (7.3) | 13 (7.3) | 11 (6.2) | 141 (79.2) | 0 (0) |
| RPC | 9 (12.5) | 14 (19.4) | 10 (13.9) | 39 (54.2) | 0 (0) |

**
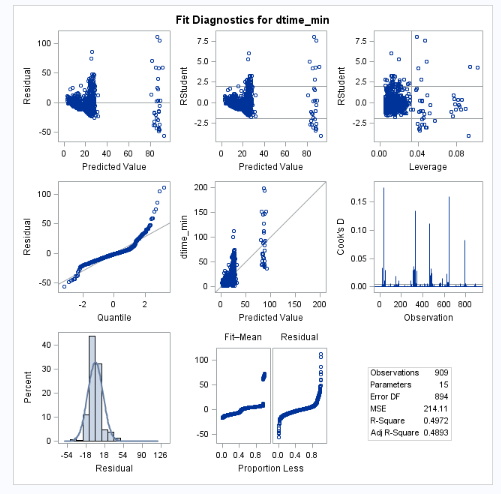
Figure A1 Fit Diagnostics for Linear Model to Examine Patient and Neighborhood Variables and One-Way Drive Time**

**Figure A2 Fit Diagnostics for Linear Model to Examine Patient and Neighborhood Variables and One-Way Drive Time**


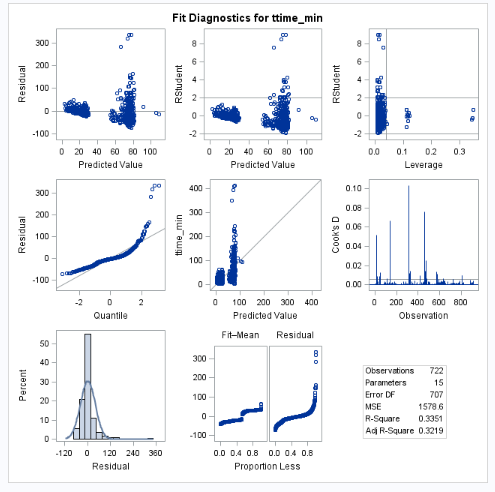


**
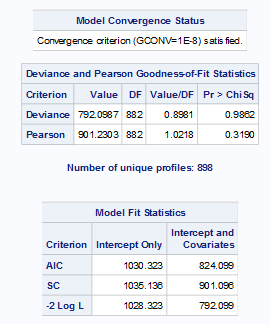
Appendix Table A3 Model Fit statistics for Logistic Model to Examine Predictors of Receiving Care at a Level 3 or RPC Birthing Hospital**
