# Supplementary material for: Proximity to risk-appropriate perinatal hospitals for pregnant women with congenital heart defects in New York state
Source: BMC Pregnancy Childbirth. 2020 Jun 1;20:338. doi: 10.1186/s12884-020-03025-4 (PMC7268637; doi:10.1186/s12884-020-03025-4)
Supplement: Supplementary file 2 — Additional file 2: Supplementary Table 1. Mutually exclusive CHD severity categories with ICD-9-CM codes. A five category CHD ICD-9-CM code classification scheme developed for the surveillance project. [file 12884_2020_3025_MOESM2_ESM.docx]

**Supplementary Table 1. Mutually exclusive CHD severity categories with ICD-9-CM codes**

| **Category** | **ICD-9-CM Code** | **Code Description** |
| --- | --- | --- |
| Severe Defect  (if cases had a severe code, regardless of presence of shunt, valve, or other codes) | 745.0 | Common Truncus |
|  | 745.1 | Transposition of the Great Arteries (TGA) |
|  | 745.10 | Complete TGA (dextro-TGA), NOS, or classical |
|  | 745.11 | DORV or incomplete TGA |
|  | 745.12 | Corrected TGA (levo-TGA) |
|  | 745.19 | TGA OS |
|  | 745.2 | Tetralogy of Fallot |
|  | 745.3 | Single Ventricle or cor triloculare |
|  | 745.6 | Endocardial Cushion Defect (AVSD) |
|  | 745.60 | Endocardial Cushion Defect (AVSD) unspecified |
|  | 745.61 | ASD-1 (primum) |
|  | 745.69 | Endocardial Cushion Defect (AVSD) other |
|  | 746.01 | Pulmonary valve atresia or absence |
|  | 746.1 | Tricuspid atresia, stenosis, or absence |
|  | 746.7 | HLHS |
|  | 747.11 | Interrupted aortic arch |
|  | 747.41 | Total anomalous pulmonary venous return (TAPVR) |
| Shunt Defect Only  (case has at least one shunt code and no valve or severe code) | 745.4 | VSD |
|  | 745.5 | ASD2 or PFO |
|  | 745.8 | Other specified defect of septal closure |
|  | 745.9 | Unspecified defect of septal closure |
|  | 747.0 | PDA |
|  | 747.10 | Coarctation of aorta |
| Valve Defect Only  (case has at least one valve code and no shunt or severe code) | 746.0 | Anomalies of pulmonary valve |
|  | 746.00 | Pulmonary valve anomaly, unspecified |
|  | 746.02 | Pulmonary valve stenosis |
|  | 746.09 | Pulmonary valve anomaly, other |
|  | 746.2 | Ebstein Anomaly |
|  | 746.3 | Aortic valve stenosis |
|  | 746.4 | Aortic insufficiency or bicuspid/unicuspid aortic valve |
|  | 746.5 | Mitral stenosis or mitral valve abnormalities |
|  | 746.6 | Mitral insufficiency |
|  | 747.3 | Anomalies of Pulmonary artery |
|  | 747.31 | Pulmonary artery atresia, coarctation, or hypoplasia |
|  | 747.39 | Anomalies of Pulmonary artery, other |
| Shunt & Valve Defects  (case has shunt and valve codes) | Variable | Variable |
| Other Defect Only  (case only has one or more codes in this category) | 745.7 | Cor biloculare |
|  | 746.8 | Other specified anomalies of the heart |
|  | 746.81 | Subaortic stenosis |
|  | 746.82 | Cor triatrium |
|  | 746.83 | Infundibular or subvalvar pulmonary stenosis |
|  | 746.84 | Obstructive anomalies of the heart |
|  | 746.85 | Coronary artery anomaly |
|  | 746.87 | Malposition of heart or apex |
|  | 746.89 | Other specified anomaly of the heart (various types) |
|  | 746.9 | Unspecified defect of the heart |
|  | 747.2 | Other anomaly of the aorta |
|  | 747.20 | Anomalies of aorta, unspecified |
|  | 747.21 | Anomaly of aortic arch |
|  | 747.22 | Atresia or stenosis of aorta |
|  | 747.29 | Other anomaly of aorta |
|  | 747.4 | Anomalies of great veins |
|  | 747.40 | Anomalies of great veins, unspecified |
|  | 747.42 | Partial anomalous venous return (PAPVR) |
|  | 747.49 | Other anomalies of great veins |
|  | 747.9 | Unspecified anomalies of circulatory system |
